# Exogenous All-*Trans* Retinoic Acid Induces Myopia and Alters Scleral Biomechanics in Mice

**DOI:** 10.1167/iovs.64.5.22

**Published:** 2023-05-23

**Authors:** Dillon M. Brown, Jianshi Yu, Praveen Kumar, Quinn M. Paulus, Michael A. Kowalski, Jay M. Patel, Maureen A. Kane, C. Ross Ethier, Machelle T. Pardue

**Affiliations:** 1 Department of Biomedical Engineering, Georgia Institute of Technology/Emory University, Atlanta, Georgia, United States; 2 Center for Visual and Neurocognitive Rehabilitation, Atlanta Veterans Affairs Healthcare System, Atlanta, Georgia, United States; 3 Department of Pharmaceutical Sciences, University of Maryland School of Pharmacy, Baltimore, Maryland, United States; 4 Department of Orthopaedics, Emory University School of Medicine, Atlanta, Georgia, United States; 5 Department of Ophthalmology, Emory University School of Medicine, Atlanta, Georgia, United States

**Keywords:** myopia, biomechanics, mouse, sclera, retinoic acid

## Abstract

**Purpose:**

Ocular all-*trans* retinoic acid (atRA) levels are influenced by visual cues, and exogenous atRA has been shown to increase eye size in chickens and guinea pigs. However, it is not clear whether atRA induces myopic axial elongation via scleral changes. Here, we test the hypothesis that exogenous atRA will induce myopia and alter scleral biomechanics in the mouse.

**Methods:**

Male C57BL/6J mice were trained to voluntarily ingest atRA + vehicle (1% atRA in sugar, 25 mg/kg) (RA: *n* = 16 animals) or vehicle only (Ctrl: *n* = 14 animals). Refractive error (RE) and ocular biometry were measured at baseline and after 1 and 2 weeks of daily atRA treatment. Eyes were used in ex vivo assays to measure scleral biomechanics (unconfined compression: *n* = 18), total scleral sulfated glycosaminoglycan (sGAG) content (dimethylmethylene blue: *n* = 23), and specific sGAGs (immunohistochemistry: *n* = 18).

**Results:**

Exogenous atRA caused myopic RE and larger vitreous chamber depth (VCD) to develop by 1 week (RE: −3.7 ± 2.2 diopters [D], *P* < 0.001; VCD: +20.7 ± 15.1 µm, *P* < 0.001), becoming more severe by 2 weeks (RE: −5.7 ± 2.2 D, *P* < 0.001; VCD: +32.3 ± 25.8 µm, *P* < 0.001). The anterior eye biometry was unaffected. While scleral sGAG content was not measurably affected, scleral biomechanics were significantly altered (tensile stiffness: −30% ± 19.5%, *P* < 0.001; permeability: +60% ± 95.3%, *P* < 0.001).

**Conclusions:**

In mice, atRA treatment results in an axial myopia phenotype. Eyes developed myopic RE and larger VCD without the anterior eye being affected. The decrease in stiffness and increase in permeability of the sclera are consistent with the form-deprivation myopia phenotype.

The eye's refractive state is jointly determined by its optical power and axial length, with deviations in either causing a refractive error that decreases visual acuity. In the healthy eye, refractive errors are minimized by a complex, homeostatic process (emmetropization) that couples regulation of axial elongation to visual cues.^1^^,^^2^ This coupling is achieved via a retinoscleral signaling cascade that propagates the sign and magnitude of visual blur from the retina, through the retinal pigment epithelium and choroid, ultimately influencing tissue remodeling in the sclera. While it is straightforward to optically correct a refractive error, such corrections do not address the underlying alterations in scleral structure, biomechanics, and eye shape, which can predispose to blinding diseases.^3^^–^^5^

For the eye size to increase, the sclera as the outermost layer must either grow (increasing in total tissue volume) or remodel (reorganizing/degrading constituent components to change tissue material properties). While the sclera grows early in life, the elongation of the eye throughout adolescence appears to be facilitated predominately by scleral remodeling that is increased in myopic eyes.^6^ The sclera from myopic eyes has reduced extracellular matrix content (primarily collagen and glycosaminoglycans) that results in a more extensible and thinner sclera than that from a control eye.^6^^,^^7^ Correspondingly, preventing or reversing such changes to the sclera has been demonstrated to protect against myopigenesis (e.g., by reinforcing the scleral collagen network^8^ or reducing collagen degradation^9^). However, scleral changes in myopigenesis are complex, with the sclera demonstrating both visually mediated^6^^,^^10^ and load-dependent remodeling.^11^^–^^13^ Further, as the eye elongates, the loading imparted to the sclera likely changes. Thus, it is not clear whether changes in scleral collagen are the cause or result of myopia. Proteoglycans (PGs)/glycosaminoglycans (GAGs) appear to be more tightly coupled to visual cues, with myopic defocus (recovery from myopia) decreasing expression and concentration of PGs/GAGs within hours.^14^ However, their influence on scleral biomechanics is still disputed.

Retinoic acid, specifically all-*trans* retinoic acid (atRA), has been proposed to be involved in the retinoscleral signaling cascade.^15^^–^^17^ atRA is produced in the retina, RPE, and choroid, and visual cues have been shown to influence ocular atRA concentrations.^18^ While the sclera does not synthesize atRA, visual cues influence the scleral expression of retinoid receptors.^15^^,^^19^^–^^21^ By coculturing choroid and sclera ex vivo, Mertz and Wallman^17^ found that the choroid is particularly leaky to atRA, and the sclera was able to concentrate atRA beyond that which would be predicted by passive diffusion. Additionally, they measured a significant effect of atRA on sulfated GAG (sGAG) synthesis in culture that is supported by in vivo work in the guinea pig,^22^ which may indicate an important scleral remodeling endpoint of retinoscleral signaling.

Emmetropization appears to function by modulating the rate of axial elongation to match optical power and thereby minimize refractive errors as the eye develops,^18^ and when emmetropization fails, the resulting eye tends to be longer, with a myopic refractive error but little difference in anterior biometry.^8^ While experimentally increasing ocular atRA concentrations causes an increase in eye size,^22^^–^^24^ there are some apparent differences in the resulting phenotype than in visually mediated models of myopia. Some differences, such as an altered anterior segment and more rapid axial elongation, could be due to the specifics of the treatment (e.g., systemic drug delivery at supraphysiologic concentrations).^8^^,^^23^^,^^24^ However, most puzzling are the findings that atRA-induced axial elongation mostly occurred without corresponding myopic refractive errors.^23^^,^^24^ Thus, it is still unclear how atRA is exerting its effects on eye size and if such changes are consistent with typical myopic axial elongation.

Here, we studied the influence of exogenous atRA on the refractive development of the mouse eye starting at 4 weeks of age. We hypothesized that increasing ocular atRA would be myopigenic, resulting in features of axial myopia such as an elongated posterior segment and altered scleral biomechanics. Scleral sGAG concentrations were measured as a possible mediator between the atRA treatment and scleral biomechanics.

## Materials and Methods

### Animals

Mice (C57BL/6J; Jackson Laboratory, Bar Harbor, ME, USA) were housed at the Atlanta Veterans Affairs Healthcare System. Animals were reared with littermates in normal lighting (12:12-hour cycles, 20–200 lux) with access to mouse chow and water ad libitum, except during the periods of atRA or vehicle feeding. Procedures were approved by the relevant Institutional Animal Care and Use Committee and adhered to the ARVO Statement for the Use of Animals in Ophthalmic and Vision Research.

### Daily Voluntary Feeding of atRA

Male C57BL/6J mice were trained to voluntarily eat sugar pellets, which were then used as a vehicle for daily oral delivery of atRA. This was a refinement in drug delivery that reduced stress to the mice.^25^ Sugar pellets (98% sucrose, water, corn syrup, maltodextrin, cornstarch; Signature Brands, LLC, Ocala, FL, USA) were formed and allowed to set at 4°C. Pellets used as a vehicle were mixed with 1% atRA (≥98% pure, cat. R2625, CAS: 302- 79-4; Sigma-Aldrich, Saint Louis, MO, USA) by weight prior to forming and setting. Pellets with atRA were stored for up to a week in a lightproof container at 4°C. Only male mice were used for these experiments since this was the first attempt to study the effects of atRA ingestion on myopigenesis, and we wanted to eliminate sources of variability that could influence our results, such as circulating estrogen, known to affect scleral biomechanical properties.^26^

Voluntary feeding was carried out by first weighing each animal and placing them into individual cages without bedding. Animals were allowed to adjust to this environment while the appropriate doses of sugar pellets or sugar + atRA were weighed out (2.5 mg pellet/g yielding 25 mg/kg atRA). Pellets were placed into each cage after wiping the cage with a paper towel. After 1 to 2 days of training (using only sugar pellets), animals voluntarily and quickly ingested the pellets (typically <5 minutes, always within 1 hour). The mice were fed between 10 am to 12 pm each day. Cages were checked every few minutes, and once a mouse ate the pellet, it was returned to its regular cage. All procedures involving atRA preparation and feeding with both atRA and sham pellets were performed in darkness or under dim, red light to prevent significant isomerization (634 nm, 0.08–7.25 cd/m^2^; Konica Minolta meter, Ramsey, NJ, USA). During feeding, mice were kept in darkness and only checked briefly (10–60 seconds) with the red light to see if the pellet had been eaten. Since the murine retina has only middle-wavelength and ultraviolet-sensitive cone photopigments, the mice were considered “blind” to the red light,^27^ and thus we do not expect physiologic effects of this dim red light during feeding periods.

To confirm that orally delivered atRA was reaching the eye, a small cohort of animals (*n* = 6) were fed atRA (RA: *n* = 3) or sugar (Ctrl: *n* = 3) for 2 weeks. After their final treatment, animals were placed in the dark for 1 to 2 hours prior to sacrifice by cervical dislocation. Eyes were enucleated, and retinas and the combined RPE, choroid, and sclera (R/C/S) were collected from each eye and stored in light-proof Eppendorf tubes at −80°C until use for retinoid quantification via liquid chromatography–tandem mass spectrometry (LC-MS/MS).

#### LC-MS/MS

Characterization of retinoids in the ocular tissues was performed as previously described.^28^ In brief, the same tissues from left and right eyes of each animal were combined and weighed prior to homogenizing in 0.9% NaCl (normal saline). A two-step acid base extraction was performed, including internal standards (RA: 4,4-dimethyl-RA; retinol, total retinyl esters: retinyl acetate).^29^ Liquid chromatography multistage tandem mass spectrometry was used to quantify RA and its isomers (9-*cis*-RA, 13-*cis*-RA, 9,13-di-*cis*-RA).^30^ HPLC-UV was used to quantify retinol and total retinyl esters.^29^^,^^31^^,^^32^ All procedures for LC-MS/MS were done under yellow light. All experimenters handling samples after tissue was collected were blinded to the identities/treatments of the animals.

### In Vivo Ocular Measurements

Mice at 4 weeks of age were fed daily with atRA or vehicle for 2 weeks (Ctrl: *n* = 14, atRA: *n* = 16) Several outcomes were measured in eyes prior to and after 1 and 2 weeks of daily feeding. Eyes were dilated with 1% topical tropicamide, and animals were anesthetized (intraperitoneal injection of ketamine: 80 mg/kg; xylazine: 16 mg/kg).^33^^,^^34^ Measurements were then made of the cycloplegic refractive error (RE) using a mouse-specific automated infrared photorefractor, corneal curvature using infrared photokeratometry, and axial ocular biometry using spectral domain optical coherence tomography (SD-OCT) (4.1-µm axial resolution; Envisu R4300; Leica Microsystems, Wetzlar, Germany).^35^^,^^36^ Registration (phase correlation) was performed on adjacent B-scans,^37^ and eye-specific spatial and temporal averaging was performed to obtain a representative B-scan with the best possible signal-to-noise ratio, as judged by the annotator. Tissue interfaces were manually delimited, from which central corneal thickness, anterior chamber depth (ACD), lens thickness, vitreous chamber depth (VCD), retinal thickness, and axial length (AL) (corneal surface to RPE) were calculated. Optical path lengths of the SD-OCT were converted to physical distances by assuming an average refractive index of 1.39.^38^^,^^39^ Less than 10% of mice were excluded due to ocular opacities at baseline. If monocular opacities were observed in the cornea or lens during follow-up, the eye was excluded from testing. No atRA-treated animals were excluded due to opacities.

In vivo measurements are presented either as values relative to the control group at each time point *t_n_* (“shifts” ≡ $x_{i}^{t_{n}}-{\bar{x}}_{Ctrl}^{\;t_{n}}$) or as a change from baseline (Δ^*t*^*x_i_*≡ $x_{i}^{\;t_{n}}-x_{i}^{t_{0}}$), where *x_i_* is the average of the two eyes from mouse *i*, ${\bar{x}}_{Ctrl}$ is the mean of the Ctrl group, and superscript *t_n_* denotes the time point. *t*_0_ denotes the baseline time point.

### Ex Vivo Endpoints

Ex vivo endpoints were assigned to each eye pseudo-randomly prior to the 1-week time point, typically assigning two different endpoints to each animal. After the 2-week treatment period, animals were sacrificed, and eyes were enucleated and processed the same day according to the chosen ex vivo endpoint, as listed below:

1. kept fresh for biomechanical measurements via unconfined compression testing (UCT),
2. frozen for quantification of total scleral sGAG/DNA content by 1,9-dimethylmethylene blue (DMMB)/PicoGreen, or
3. fixed for semiquantitative immunohistochemistry (IHC).

**Figure 1.**
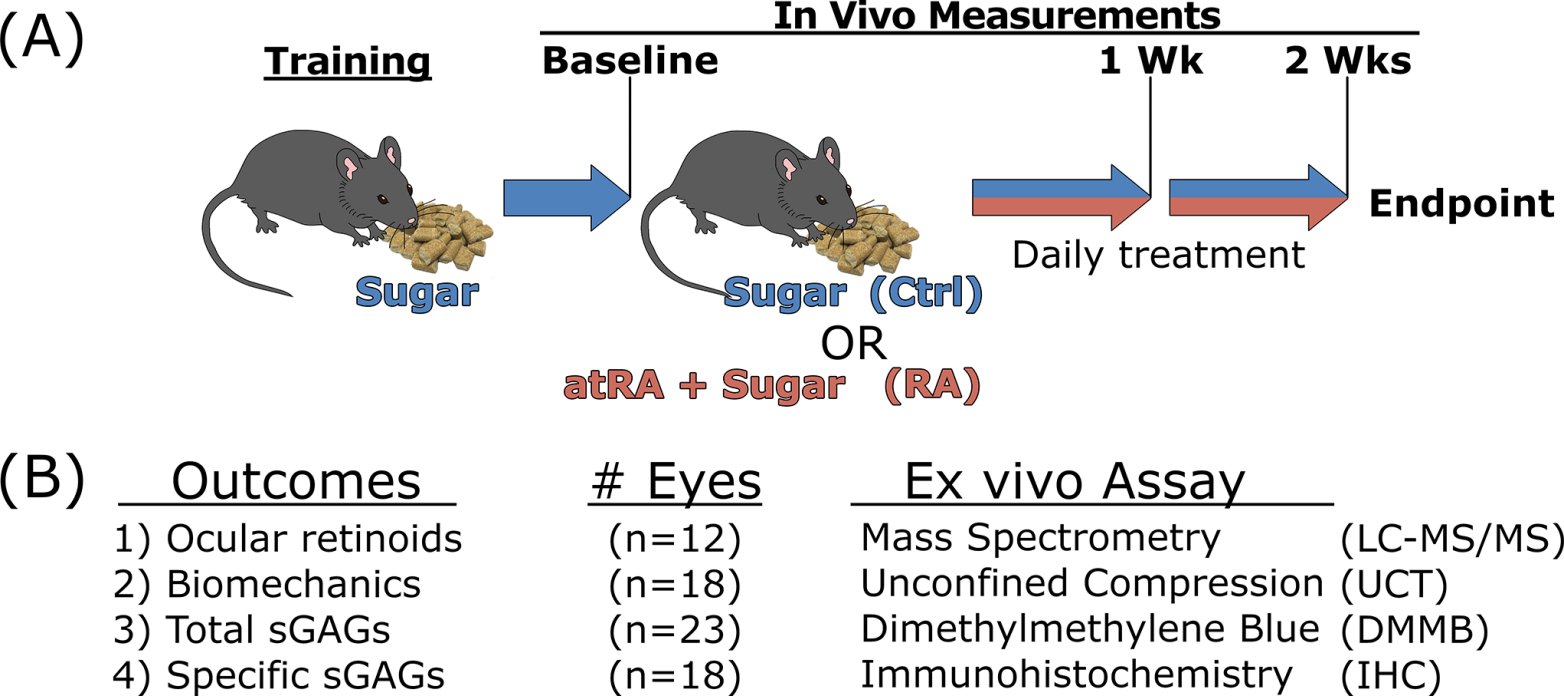
Experimental study design. (**A**) In vivo measurements were made at baseline, after which animals were either assigned to daily feeding of atRA in sugar pellets (RA; 25 mg/kg) or sugar pellets only (Ctrl). Treatments were maintained for 2 weeks, with body mass recorded daily and ocular measurements made weekly (three times total). (**B**) The outcomes studied are presented alongside their associated ex vivo assays and the number of eyes sampled.

In total, 59 eyes from 30 animals were studied across the three ex vivo endpoints, in addition to 12 eyes used for confirming oral atRA reached the ocular tissues (Fig. 1).

#### UCT

Eyes were stored in phosphate-buffered saline (PBS) at 4°C after enucleation and scleral biomechanical properties were characterized the same day as previously described.^40^ Posterior eye cups were obtained as described above for LC-MS/MS measurements (under normal lighting). The retina was removed from the posterior eye cup. Four cuts were made to flatten the posterior eye, and the choroid and RPE were removed. A biopsy punch was used to obtain 1-mm diameter cylindrical samples from the posterior region of the scleral shell (∼0.5 mm from the optic nerve). A small amount of graphite powder was applied to samples to minimize friction between samples and compressive platens. Samples were then placed into a 37°C PBS fluid bath, in which biomechanical testing was carried out.

A compression testing apparatus designed for small samples (Microsquisher; CellScale, Waterloo, ON, Canada) was used to perform unconfined compression stress relaxation experiments on scleral samples. A tare load (500 µN) was applied to flatten the samples and ensure samples were in contact with the platens. After equilibrating, three compressive strain steps were incrementally applied (5% strain per step, 15% maximum compressive strain). At each step, strain was rapidly applied and maintained until stress relaxed and equilibrated, after which the next step was started. The biphasic conewise linear elastic model was independently fit to each step, whose parameters include the aggregate tensile modulus H_+A_ (i.e., in-plane tensile stiffness) and hydraulic conductivity k (i.e. permeability to water).^40^ Note that it is possible to obtain in-plane scleral tensile mechanical properties from this compressive scleral testing paradigm, since through-plane scleral compression leads to orthogonal (in-plane) tissue expansion. One estimate of each parameter was obtained at each of the three strain steps, yielding three measurements of each parameter per sample over the test.

Due to the variability in the strain applied at each step and the dependence of the material properties on compressive strain, both the raw values and estimated marginal means obtained from the fitted statistical model are reported, in which strains are adjusted to exactly the target strains (5%, 10%, and 15%).

#### DMMB and PicoGreen Assays

Eyes were stored in PBS at 4°C until dissection the same day, following the same procedures described for unconfined compression. Isolated sclerae were dehydrated overnight in a vacuum desiccator and weighed three times on an analytical balance (XSR205DU; Mettler Toledo, Columbus, OH, USA), with the average being taken as the dry mass of the sclera. The isolated sclerae were stored at −80°C until use.

Total scleral sGAG and DNA content were quantified via spectrophotometric assays, the DMMB assay and PicoGreen assay, respectively.^28^ In brief, samples were thawed and digested in 200 µL papain solution (0.1 M sodium acetate [anhydrous], 10 mM cysteine hydrochloride, 50 mM EDTA, pH 6, 2% v/v papain), distributing the digestate between the two assays. For the DMMB assay, absorbance was read at 525 nm immediately following the addition of the DMMB dye mixture (8 mg DMMB, 2.5 mL ethanol, 1 g sodium formate, 1 mL formic acid, and 496.5 mL deionized water). To measure DNA, the PicoGreen working reagent was added and absorbance was read using 480-nm excitation and 520-nm emission.

Concentrations of each were then determined by comparing absorbances to a standard curve. Absorbances from the DMMB assay were compared to shark cartilage chondroitin sulfate (working range 0–25 µg sGAG/µL, eight measured standards; Sigma C4384). Absorbances from the PicoGreen assay were compared to Lambda DNA stock (working range 0–4 µg DNA/µL, eight measured standards; Sigma cat. SD0011; Thermo Fisher Scientific, Waltham, MA, USA). Both assays were performed in triplicate, with the average of the three readings taken as the final value. Scleral sGAG content is reported as a mass fraction (µg sGAG/mg sclera) and as a mass ratio (µg sGAG/µg DNA) to account for differences in quantity of collected tissue.

#### IHC

Eyes were enucleated and immersion fixed in buffered zinc formalin (Z-Fix; Anatech LTD, Battle Creek, MI, USA) for 1 hour immediately following sacrifice. Extraocular tissue, cornea, and lens were removed after fixation. Prior to cryosectioning, eyes were allowed to equilibrate in 30% sucrose solution overnight, following which they were embedded in optimal cutting temperature medium and frozen on the Peltier cooling element of a cryostat (CM1850; Leica Microsystems). Sagittal sections were taken through the optic nerve head (10 µm), applied to charged slides (Superfrost Plus; Thermo Fisher Scientific), and stored at −20°C.

A monoclonal primary antibody (clone 2B6, cat. 1042009, lot: S1605002; MD Biosciences, Oakdale, MN, USA) was used to stain specific sGAGs. 2B6 is specific to neoepitopes generated by chondroitinase digestion of chondroitin-4-sulfate (C-4-S) and dermatan sulfate (DS) but not chondroitin-6-sulfate (C-6-S). C-4-S and DS were separately labeled by treating tissue sections with one of two chondroitinases: either chondroitinase AC (ChAC), which specifically deglycosylates chondroitin A (C-4-S) and chondroitin C (C-6-S), or chondroitinase B (ChB), which targets chondroitin B (DS) (both from *Flavobacterium heparinum*; Sigma-Aldrich). Thus, one antigen site recognized by 2B6 will be exposed per C-4-S chain when tissue is treated with ChAC; similarly, one site recognized by 2B6 will be exposed per DS chain when treated with ChB.

Tissue was prepared for immunostaining by first washing the samples with Tris-buffered saline with 0.1% Triton X-100 (TBS-T) to remove embedding medium, and then samples were treated with chondroitinase (3 hours at 24°C; ChB, ChAC, or buffer only; 0.5 U/mL, 8.0 pH). Sections were then permeabilized (10 minutes at 24°C; 0.5% Triton X-100 in TBS), blocked (45 minutes at 24°C; 10% normal goat serum, 1% bovine serum albumin in TBS-T), and incubated with primary antibody overnight (18 hours at 4°C; mouse monoclonal antibody 2B6 in blocking buffer, 1:100). The next day, sections were washed and incubated with secondary antibodies (45 minutes at 24°C in darkness; 1:500; Alexa Fluor 647–conjugated goat anti-mouse antibodies in blocking buffer, cat. A-21244; Invitrogen, Carlsbad, CA, USA). Samples were washed, treated with DAPI (NucBlue Fixed Cell Stain ReadyProbes DAPI, cat. R37606; Invitrogen), washed, and coverslipped with antifade medium (Prolong Diamond Antifade Medium, cat. P36961; Invitrogen). Staining was done using the same batch of working reagents on all slides in parallel, and each slide had sections from the same eye treated with each of the chondroitinase conditions (ChB treated, ChAC treated, and buffer-only control).

A confocal microscope (Nikon A1R; Nikon, Tokyo, Japan) with an apochromatic 20× objective lens was used to image immunolabeled tissue, keeping all settings the same across all samples. Three images (600 × 600 µm each, two channels, 12 bits per channel) were taken per section, centered on the optic nerve head as a reference location. Semiautomated macros in FIJI^41^ were used to stitch, mask, threshold, and quantify images. The sclera was manually masked from the adjacent choroid and the region directly surrounding the optic nerve. For regional analysis, the masked regions were split at the optic nerve to divide the superior and inferior sclera.

On each slide, average pixel intensities within the masked sclera were calculated for a ChB-treated section, a ChAC-treated section, and a buffer-only section. Normalized fluorescence is reported as a semiquantitative measure of the specific sGAGs (DS = intensity of ChB-treated section/intensity of buffer-only; C-4-S = intensity of ChAC-treated section/intensity of buffer only) and is related to the number of GAG attachment sites on proteoglycans.

### Statistical Analysis

Each outcome measure was analyzed by constructing a generalized linear mixed model reflecting the experimental design. Outcomes were assigned a Gamma error distribution with a log link when this led to reasonable predictions (e.g., strictly positive quantities) and based on analysis of the model residuals (Supplementary Table S1). Significance of fixed effects was calculated by performing likelihood ratio tests between the full model and null models, each lacking one of the fixed-effect terms. Random intercepts were included to account for intersample correlation, such as multiple measurements of the same animals/eyes. Fitting, contrasts, and *P* value calculations were done using the “lme4” and “emmeans” packages in R (version 4.1.1).^42^^,^^43^ The significant α threshold was set to 0.05, and when multiple contrasts were run, adjusted *P* values are reported (multivariate *t* method).^44^

## Results

### Orally Delivered atRA Was Well Tolerated by Mice

**Figure 2.**
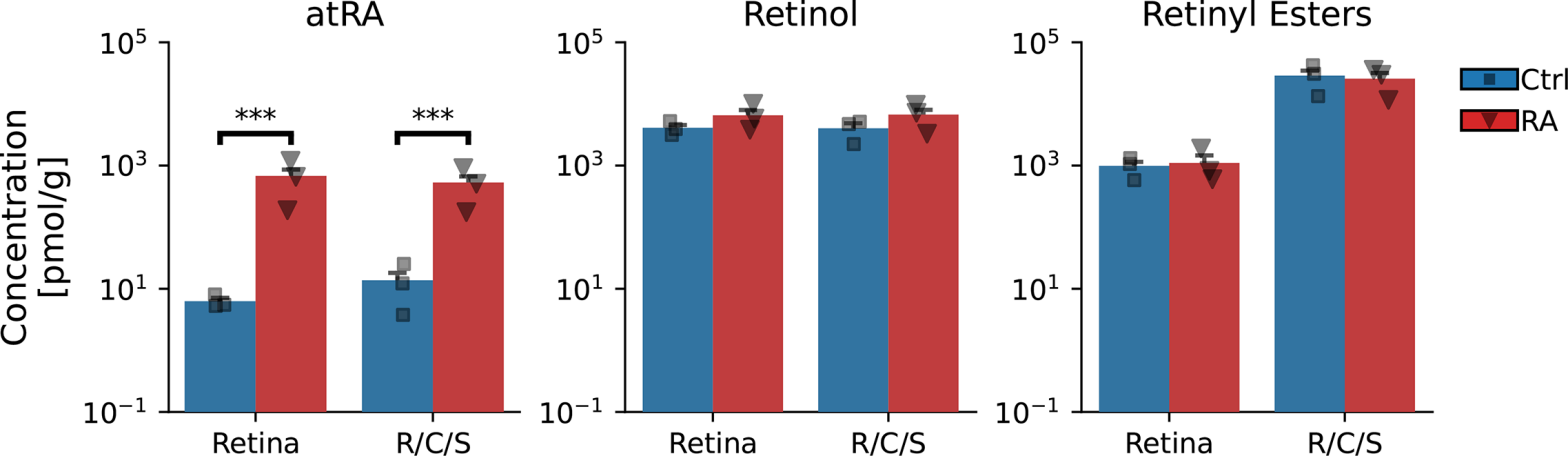
Oral delivery of atRA significantly increased the concentration of atRA in the retina and R/C/S without influencing levels of other retinoids. Similar tissues from the two eyes from each animal were grouped and measured together. R/C/S, combined retina, choroid, and sclera; Ctrl, vehicle-treated group (*n* = 3 animals/6 eyes); RA, atRA-treated group (*n* = 3 animals/6 eyes). *P < 0.05, **P < 0.01, ***P < 0.001.

Animals treated with atRA daily for 2 weeks displayed no obvious changes in behavior or health; no animals were lost due to treatment. Animals consumed food/water normally, and treatment did not affect the normal body mass development (interaction: treatment/duration, *P* = 0.06, Supplementary Fig. S1). Animals voluntarily ingested the drug for the entire treatment period. Oral ingestion of atRA significantly increased ocular atRA levels (atRA versus Ctrl: *P* < 0.001 for both retina and combined R/C/S) without affecting the levels of other retinoids (Fig. 2). On average, isomerization of atRA to all measured *cis* forms (combined) was approximately 7%, which is the amount expected when atRA is in the presence of biological tissue matrix,^45^ and there were no signs of significant isomerization in the internal standards.

### Increasing Ocular atRA Induced Axial Myopia in the Mouse

**Figure 3.**
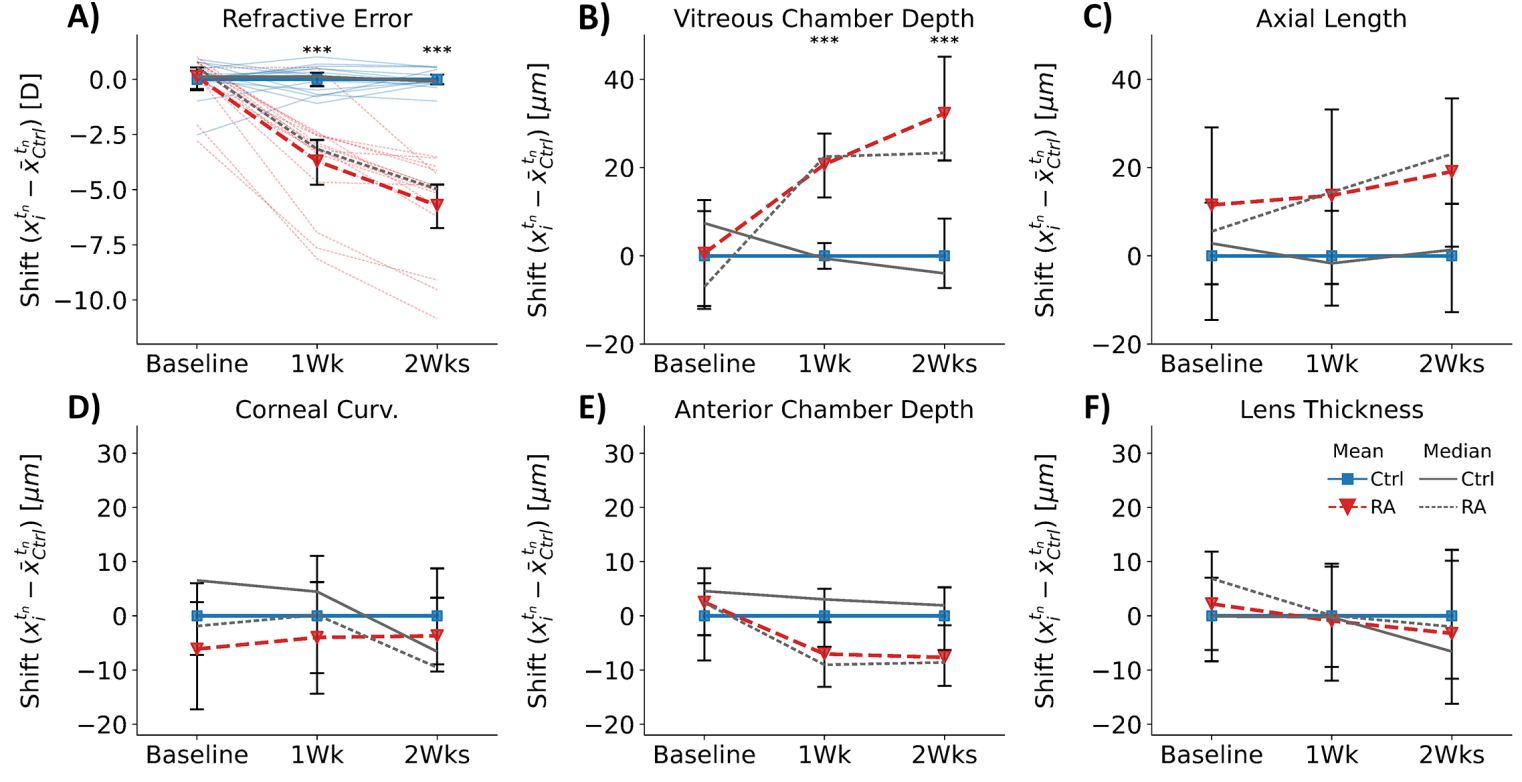
Treatment with atRA caused significant changes in refractive state and ocular biometry. Plots show the “shifts” of each outcome (shift = $x_{i}^{t_{n}}-{\bar{x}}_{Ctrl}^{\;t_{n}}$), where $x_{i}^{t_{n}}$ represents the measured value in animal *i* at time point *t_n_* and ${\bar{x}}_{Ctrl}^{\;t_{n}}$ is the mean of the control group at time point *t_n_*. (**A**) Control and atRA-treated animals had very similar refractive errors at baseline prior to treatment. One and 2 weeks of daily atRA caused significant myopia to develop. (**B**) VCD increased with atRA feeding, consistent with a myopic refractive error. (**C–F**) Other measurements of optical biometry were unaffected by atRA, including those in the anterior chamber. Individual animals are shown with *fainter lines* in **A**. *Solid blue* (*gray*) *lines* are the mean (median) of the control group, and *dashed red* (*gray*) lines are the mean (median) of the atRA-treated group. *Error bars* show the 95% confidence intervals. Ctrl, vehicle-treated group (*n* = 14 animals); RA, atRA-treated group (*n* = 16 animals). *P < 0.05, **P < 0.01, ***P < 0.001, adjusting for deviations in age and multiple comparisons.

Prior to treatment, animals in both groups had similar refractive errors (RE shift; 0.12 ± 1.1 diopters [D], *P* = 0.72). Treatment with atRA for both 1 and 2 weeks caused animals to develop significant myopic shifts in refractive error (RE shift; 1 week: −3.7 ± 2.2 D, *P* < 0.001; 2 weeks: −5.7 ± 2.2 D, *P* < 0.001). Every animal treated with atRA was relatively myopic compared to the mean of the littermate controls after 2 weeks (Fig. 3A).

Measurements of AL and VCD were consistent with the development of refractive errors. At baseline, neither VCD nor AL were different between treatment groups (VCD: *P* = 0.72, AL: *P* = 0.55, Supplementary Table S2). VCD decreased with age in both groups but less in the atRA-treated animals than controls (VCD Δ^*t*^*x_i_*; 1 week: Ctrl: −25.2 ± 24.2 µm, RA: −2.2 ± 25.8 µm, *P* < 0.001; 2 weeks: −45.1 ± 32.1 µm, RA: −18.2 ± 37.3 µm, *P* < 0.001, Supplementary Fig. S2), resulting in atRA-treated animals having deeper vitreous chambers at both time points (VCD shift; 1 week: +20.7 ± 15.1 µm, *P* < 0.001; 2 weeks: +32.3 ± 24.8 µm, *P* < 0.001; Fig. 3B). The VCD decreases with age due to the growth of the lens overtaking the rate of elongation. In other words, because the lens development is not affected by treatment, the shift in VCD is consistent with excess posterior elongation of the eye.

A similar but more variable trend was observed in axial length (Fig. 3C). While the mean axial lengths were not significantly different at any time point (AL shift; 1 week: 13.7 ± 42.5 µm, *P* = 0.42; 2 weeks: 19.1 ± 34.5 µm, *P* = 0.14), atRA-treated eyes elongated more than control eyes after both 1 and 2 weeks (AL Δ^*t*^*x_i_*; 1 week: Ctrl: 82.6 ± 14.7 µm, RA: 90.9 ± 17.5 µm, *P* = 0.024; 2 weeks: Ctrl: 132.1 ± 15.1 µm, RA: 145.0 ± 16.6 µm, *P* < 0.001).

Treatment with atRA did not cause any significant shifts in anterior eye biometry outcomes that influence optical power (anterior corneal curvature, corneal thickness, lens thickness, anterior chamber depth) (Supplementary Table S2). However, atRA treatment had a minor but significant effect on the development of the anterior chamber depth. Specifically, the anterior chamber depth increased over time in both groups but less so in the atRA-treated animals (main effects across age, ACD Δ^*t*^*x_i_*; 1 week: Ctrl: +19.9 ± 7.5 µm, RA: +11.1 ± 8.8 µm, *P* < 0.001; 2 weeks: +35.7 ± 9.1 µm, RA: +26.7 ± 7.1 µm, *P* < 0.001, Supplementary Fig. S2E), but this did not result in a significant shift at either time point (interaction effect, ACD shift; baseline: 2.5 ± 11.7 µm, *P* = 0.97; 1 week: −7.0 ± 12.8 µm, *P* = 0.29; 2 weeks: −7.7 ± 12.0 µm, *P* = 0.22, Fig. 3E).

### Scleral Stiffness and Permeability Were Both Altered as a Result of Increased Ocular atRA

**Figure 4.**
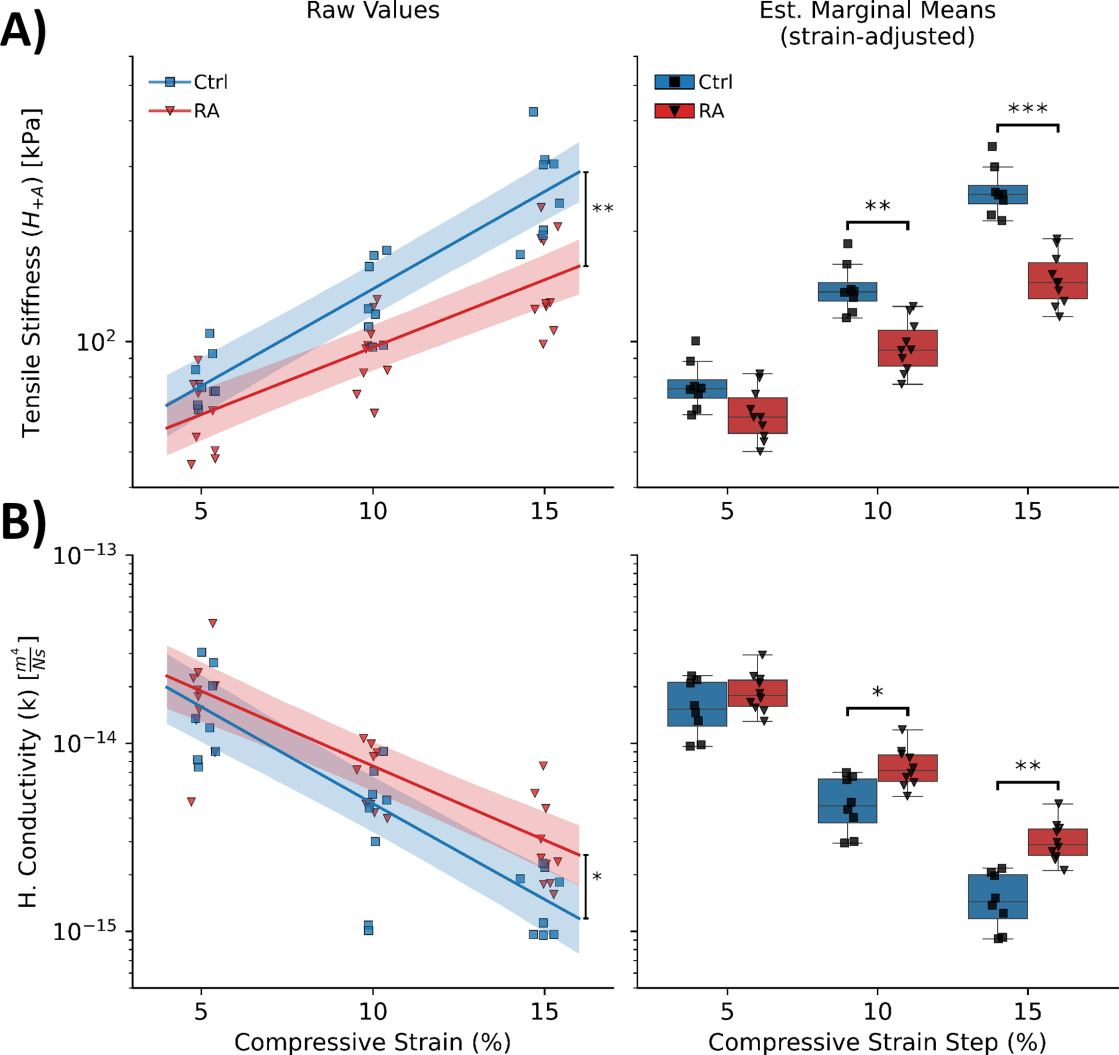
Scleral biomechanics were altered in response to atRA treatment. (**A**) In-plane aggregate tensile modulus (H_+A_) (i.e., stiffness) and (**B**) hydraulic conductivity (k) (i.e., permeability) were altered due to treatment. Plots show the raw values obtained from fitting the poroelastic material model, regressions, and 95% credible intervals obtained from the statistical model (*left*) and the estimated marginal means obtained from the statistical model (*right*), both plotted as a function of the applied compressive strain. Ctrl, vehicle-treated group (*n* = 8 animals/eyes); RA, atRA-treated group (*n* = 10 animals/eyes). *P < 0.05, **P < 0.01, ***P < 0.001.

Scleral biomechanical properties were altered by atRA feeding. Averaged across all strain steps, sclerae from atRA-treated animals were less stiff (atRA: 104 ± 47 kPa, Ctrl: 160 ± 96 kPa, main effect: treatment, *P* < 0.001) and more permeable (atRA: 0.96E-14 m^4^/Pa*s, Ctrl: 0.73E-14 m^4^/Pa*s, main effect: treatment, *P* = 0.02) (Fig. 4). When compared to the mean of the control group at the same strain step, the sclerae from atRA-treated animals were ∼30% less stiff and ∼60% more permeable compared to control animals (Supplementary Table S4).

These differences were more pronounced with increasing compressive strain (interactions: stiffness/strain: *P* < 0.001, permeability/strain: *P* = 0.03). Specifically, with increasing compressive loads, sclerae from atRA-treated animals stiffened less (*P* < 0.001) and permeability decreased less (*P* = 0.03) than was the case for control sclerae (Fig. 4). For example, sclerae from atRA-treated eyes were approximately half as stiff and twice as permeable than those from control eyes at 15% compressive strain (Supplementary Table S4). Using the fitted statistical model to estimate properties at 0% compressive strain, there were no differences between atRA and control eyes in either outcome.

### Scleral GAGs and DNA Content Were Not Measurably Altered With atRA Treatment

**Figure 5.**
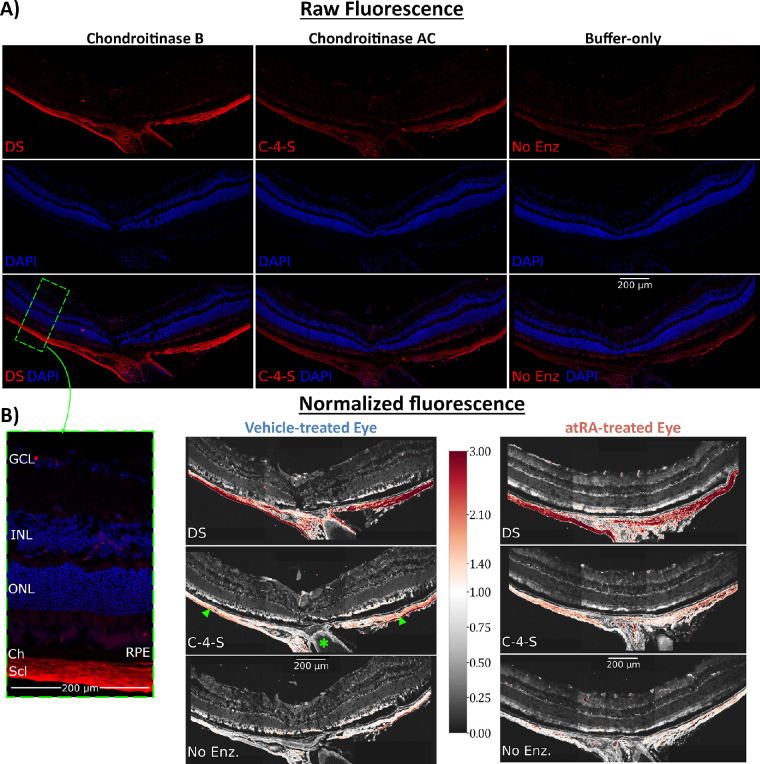
Effect of oral atRA on immunostaining of specific scleral sGAGs. (**A**) Representative immunostaining of the posterior eye subjected to the different chondroitinase treatments. Staining was minimal in the absence of chondroitinase during incubation (*top right*). When samples were incubated with either ChAC (*top middle*) or ChB (*top left*), labeling increased, primarily in the sclera. The *green dashed rectangle* outlines a 200-µm-wide region and its magnified/annotated view. DAPI labeling in the sclera is present but difficult to see due to the exposure setting used. Image exposure was chosen so that DAPI labeling in the nuclei-dense retina did not saturate the blue channel, leading to dim DAPI labeling in the relatively nuclei-sparse sclera. (**B**) From the immunostaining in (**A**), normalized fluorescence is obtained for ChB and ChAC conditions by normalizing to the average intensity in the sclera of the buffer-only condition. There were no quantifiable differences between the control and atRA-treated animals. *Red pixels* indicate increased staining, *white* indicates no change, and *gray* indicates decreased staining relative to the sclera of the same eye's “buffer-only” treatment, as shown in the color bar. *Arrowheads* label the sclera; the *asterisk* denotes the optic nerve.

No effect of atRA was seen on the DNA content of the total sclera, indicating no large influence on the proliferation of resident cells (Ctrl: 1.19 ± 0.16 µg DNA/mg sclera, RA: 1.29 ± 0.21 µg DNA/mg sclera). Two measurements of sGAGs in the sclera were performed: total concentration in the sclera (via DMMB assay) and fluorescence intensity of immunolabelling in the posterior sclera (via IHC as a semiquantitative proxy for number of sGAG attachment sites). Neither sGAG concentration nor fluorescence intensity were affected by atRA treatment (Fig. 5, Table). An additional regional analysis was performed on the IHC images, in which the regions superior and inferior to the optic nerve were analyzed separately, and no regional differences were observed (Supplementary Fig. S3).

**Table.** Biochemical Makeup of the Sclera Was Not Measurably Changed by atRA Treatment

Group	Body Weight, g	Sclera Mass (Dry), µg	DNA Concentration, µg/mg	GAG Concentration, µg/mg	GAG/DNA, µg/µg	C-4-S, AU	DS, AU
Ctrl	22.89 ± 1.65 (9)	140.22 ± 21.94 (9)	1.19 ± 0.16 (9)	7.18 ± 2.10 (9)	5.97 ± 1.30 (9)	1.48 ± 0.28 (7)	3.69 ± 1.31 (7)
RA	20.21 ± 2.33 (14)	135.33 ± 16.93 (14)	1.29 ± 0.21 (14)	8.70 ± 1.98 (14)	6.81 ± 1.43 (14)	1.52 ± 0.32 (11)	3.91 ± 1.42 (11)

Tabulated quantities are mean ± standard deviation (number of eyes). Scleral DNA was quantified by the PicoGreen assay, and sGAGs were quantified by both the DMMB assay (total) and immunostaining of chondroitinase-treated samples (specific sGAGs). Ctrl: vehicle-treated group (*n* = 14 animals); RA: atRA-treated group (*n* = 16 animals). AU, arbitrary units (normalized fluorescence).

## Discussion

The present study demonstrates a causal link between increased ocular atRA levels and myopic axial elongation in the mouse. Systemic delivery of atRA specifically increased the concentration of atRA in the eye, without increasing other retinoic acid isomers, and resulted in mice developing myopic refractive errors. Treatment with atRA minimally affected the biometry of the anterior eye but led to significantly increased axial elongation and correspondingly longer vitreous chambers than control animals, indicating an elongation of the posterior eye that is consistent with axial myopia in other species. We have also established that the myopic phenotype induced by atRA is accompanied by altered scleral biomechanics, with the sclera becoming more extensible and permeable, an effect consistent with that seen in visually mediated myopia in other species,^10^^,^^46^^–^^48^ including our previous report in the mouse.^28^ The overall impact of atRA on the eye appeared to be limited entirely to the posterior eye, except for a minor effect on anterior chamber depth that was also observed to occur in chickens.^24^ We did not identify changes in scleral sGAGs or obvious signs of systemic or ocular toxicity. Together, these findings support a role for atRA in myopigenic retinoscleral signaling by influencing both axial length and scleral biomechanics similarly to visually mediated myopigenesis.

### atRA Causes Axial Myopia in the Mouse

The results of this study are consistent with the results of previous studies in guinea pigs and chickens^22^^–^^24^ demonstrating that experimentally elevating atRA levels causes an increase in eye size; however, it is not clear by what mechanism this occurs. In the studies by McFadden et al.,^23^ atRA caused increases in eye size but did not induce refractive errors, indicating that the optical power of the eyes was also affected in a manner that kept the focal point of light on the retina. Additional significant changes uncharacteristic of axial myopia were observed by McFadden et al.^24^ (e.g., a thinner lens and shorter anterior chamber, even with atRA doses as small as 2 mg/kg). While Li et al.^22^ reported myopic refractive errors, axial elongation, and changes in scleral proteoglycans in response to atRA in guinea pigs, other ocular biometry was not reported, and scleral proteoglycans were not quantified,^22^ making comparison to the other studies difficult. Thus, most previous studies suggest that atRA causes the eye to enlarge in a manner disparate from what occurs in axial myopia.

In agreement with previous studies, we have demonstrated a causal connection between ocular atRA and the size of the eye.^23^^,^^24^ However, the present study has shown that increasing ocular atRA levels caused mice to develop myopic refractive errors; notably, eyes elongated more over the treatment period and had longer vitreous chambers on average than the control animals, and the anterior segment was only minimally affected. Additionally, the biomechanics of the sclera were significantly altered, resulting in a biomechanical phenotype in close agreement with the outcomes of form-deprivation models of myopia^28^; specifically, atRA caused the sclera to become more extensible and more permeable.

While we measured no effect of atRA on lens thickness, we did observe a mild shortening of the anterior chamber similar to that observed in the guinea pig.^23^ McFadden et al.^23^ speculated that the effect on ACD could be the result of a flattened cornea; however, we observed no changes in the anterior corneal curvature with atRA treatment. Whatever the cause of the change in ACD, the effect was relatively small when compared to the axial elongation. Estimates from simple ray tracing would suggest the ∼8-µm decrease in ACD seen here would be responsible for <1 D of myopia.^49^ In contrast, the ∼30-µm longer vitreous chamber is estimated to cause 6 D of myopia in the mouse, which closely agrees with the measured ∼–5.7 D refractive error.^50^ Thus, while atRA may have a minor impact on the anterior ocular tissues, the effects in the mouse are predominately in the posterior eye and characteristic of myopic axial elongation.

The present study has significant phenotypic differences from those reported previously by McFadden et al.,^23^ and comparison could be informative. Two primary methodologic differences between the present study and those of McFadden et al.^23^ are in the means of drug delivery (voluntary feeding here versus gavage by McFadden et al.^23^) and the age of the animals at the onset of treatment. While gavage and feeding both deliver the drug systemically via the gastrointestinal tract, it has been previously shown that there are differences in the effect of atRA when the two methods are compared directly.^25^ Perhaps more relevant to myopigenesis is the choice of age of the animals. Here, treatment was initiated at 4 weeks of age, when the mouse eye has already reached 90% to 95% of its mature size but is still emmetropizing.^49^^,^^51^ In the studies by McFadden et al.,^23^^,^^24^ both guinea pigs and chickens were treated starting at 1 week of age, when the eye is still elongating rapidly and is only approximately 60% to 80% of its mature size.^52^^,^^53^ Considering that atRA is centrally involved in developmental processes in many organs, influencing the transcription of around 100 genes and displaying precise spatiotemporal regulation,^54^ it is not unreasonable to speculate that atRA may be involved in modulating both eye growth and myopigenic axial elongation, with the relative contribution to these processes depending on the age of the animal. However, to date, no studies have directly explored this hypothesized dual role.

### Form Deprivation Compared to atRA-Induced Myopigenesis in the Mouse

We have previously reported that form deprivation in the mouse results in altered scleral biomechanics and decreased scleral sGAGs levels.^28^ Specifically, after 3 weeks of form deprivation, mice developed 4.1 D of myopia, and the sclera was 40% more extensible and 140% more permeable compared to the contralateral control eyes. Here, daily atRA treatment for 2 weeks caused 5.7 D of myopia and an average 30% and 60% increase in scleral extensibility and permeability compared to the control group. Considering the difference in how these percentages were calculated (pairwise within each animal in the form-deprivation study, compared to mean of the control group in the current study) and the fact that the minor effects of atRA on ACD may be responsible for ∼1 D of refractive myopia, the degree of myopigenesis and associated scleral biomechanical changes agree reasonably well.

However, whereas we previously measured a decrease in C-4-S and DS in the sclera of form-deprived eyes, here, using identical methodologies, we saw no measurable change in scleral sGAG content with atRA treatment. While this difference could be indicative of disparate remodeling and myopigenesis mechanisms, it could also be an artifact of the treatment. One obvious difference between form deprivation and atRA as delivered in this study is the different temporal characteristics of the stimuli. To induce form-deprivation myopia, a diffuser lens is maintained over the eyes of animals, which presumably yields a roughly steady-state retinoscleral signal. However, the bioavailability of orally ingested atRA will vary with time, as it is uptaken and eventually metabolized. In chickens, atRA delivery caused a day of rapid growth before a rebound the following day.^24^ Both scleral biomechanics and sGAG synthesis have been demonstrated to respond rapidly to visual stimuli, on the time scale of hours to days.^55^ Thus, it is possible that by the time animals were sacrificed (∼24 hours after their last dose of atRA), sGAG content had rebounded back to or above the control level.

However, there is also precedent in the literature showing that periods of myopigenic stimuli followed by periods of normal vision can prevent certain aspects of the myopic phenotype, from axial elongation to choroidal changes.^17^ The bolus delivery of atRA in this study possibly mimics this type of signaling to some degree, and perhaps sGAGs are not reduced unless scleral atRA is maintained above normal levels for longer periods of time. Further studies on the short-term effects of atRA are warranted to help elucidate these potential confounders and better understand the time scales of the scleral response to atRA.

### Altered Scleral Biomechanics Without Altered sGAGs

GAG content has been demonstrated to influence the hydraulic conductivity of biological tissues. Consistent with such data, we previously reported that both scleral hydraulic conductivity and C-4-S levels changed in the mouse model of form-deprivation myopia.^28^ However, in the present study, treatment with atRA caused changes to the hydraulic conductivity without a measurable effect on sGAG content. One explanation for this apparent “decoupling” between conductivity and sGAG levels may be that another unmeasured scleral constituent that influences the hydraulic conductivity was altered, such as another GAG (e.g., heparin sulfate) or the architecture of the collagen network was changed. A second possible explanation is that the physical distribution/configuration of sGAGs changed at the cellular level without a change in overall scleral sGAG concentration, since such changes are known to affect hydraulic conductivity in other tissues.^56^^,^^57^ In particular, if sGAG configuration were to be affected by the biomechanical environment (strain levels), this could also explain why neither hydraulic conductivity nor stiffness estimated at 0% compressive strain were significantly different between the atRA-treated and control sclera. Examination of glycosaminoglycan synthesis would also be a useful approach for future experiments, since such information may be a more sensitive indicator of changes in scleral composition^58^^,^^59^ and complements information about total scleral GAG content, which influences scleral biomechanics and elongation.

## Conclusion

We have demonstrated that orally delivered atRA is highly myopigenic in mice, causing myopic refractive errors. In contrast to previous studies, the anterior eye was largely unaffected, whereas the posterior eye was significantly elongated, both consistent with a typical axial myopia phenotype. For the first time, the effect of atRA on scleral biomechanics was studied, where atRA treatment led to a more extensible and permeable sclera, comparable to what is seen in the sclerae of form-deprived eyes. This study supports a role for atRA in scleral-mediated myopia development, with scleral changes similar to that found in visually mediated myopigenesis. Future work examining the effects of atRA, both in animals of different ages and studying effects at different times after atRA administration, is warranted to better understand the myopic phenotype.

## Supplementary Material

Supplement 1

## References

[1] Flitcroft DI . Is myopia a failure of homeostasis? *Exp Eye Res*. 2013; 114: 16–24. 23454097 10.1016/j.exer.2013.02.008

[2] Troilo D, Smith EL, Nickla DL, et al. IMI—report on experimental models of emmetropization and myopia. *Invest Ophthalmol Vis Sci*. 2019; 60(3): M31–M88. 30817827 10.1167/iovs.18-25967 PMC6738517

[3] Saw SM, Gazzard G, Shih-Yen EC, Chua WH. Myopia and associated pathological complications. *Ophthalmic Physiol Opt*. 2005; 25(5): 381–391. 16101943 10.1111/j.1475-1313.2005.00298.x

[4] Shen L, Melles RB, Metlapally R, et al. The association of refractive error with glaucoma in a multiethnic population. *Ophthalmology*. 2016; 123(1): 92–101. 26260281 10.1016/j.ophtha.2015.07.002 PMC4695304

[5] Lakawicz JM, Bottega WJ, Fine HF, Prenner JL. On the mechanics of myopia and its influence on retinal detachment. *Biomechanics Model Mechanobiol*. 2020; 19(2): 603–620. 10.1007/s10237-019-01234-1 31650370

[6] Harper AR, Summers JA. The dynamic sclera: extracellular matrix remodeling in normal ocular growth and myopia development. *Exp Eye Res*. 2015; 133: 100–111. 25819458 10.1016/j.exer.2014.07.015 PMC4379420

[7] Boote C, Sigal IA, Grytz R, Hua Y, Nguyen TD, Girard MJA. Scleral structure and biomechanics. *Prog Retin Eye Res*. 2020; 74: 100773. 31412277 10.1016/j.preteyeres.2019.100773 PMC7187923

[8] Grytz R. Scleral remodeling in myopia. In: Roberts CJ, Dupps WJ, Downs JC, eds. *Biomechanics of the Eye*. Amsterdam, Netherlands: Kugler; 2018: 383–403.

[9] Zhao F, Zhou Q, Reinach PS, et al. Cause and effect relationship between changes in scleral matrix metallopeptidase-2 expression and myopia development in mice. *Am J Pathol*. 2018; 188(8): 1754–1767. 29803830 10.1016/j.ajpath.2018.04.011

[10] Grytz R, Siegwart JTJ. Changing material properties of the tree shrew sclera during minus lens compensation and recovery. *Invest Ophthalmol Vis Sci*. 2015; 56(3): 2065–2078. 25736788 10.1167/iovs.14-15352 PMC4373544

[11] Girard MJA, Suh JKF, Bottlang M, Burgoyne CF, Downs JC. Biomechanical Changes in the Sclera of Monkey Eyes Exposed to Chronic IOP Elevations. *Invest Ophthalmol Vis Sci*. 2011; 52(8): 5656–5669. 21519033 10.1167/iovs.10-6927 PMC3176060

[12] Grytz R, Girkin CA, Libertiaux V, Downs JC. Perspectives on biomechanical growth and remodeling mechanisms in glaucoma. *Mechanics Res Commun*. 2012; 42: 92–106. 10.1016/j.mechrescom.2012.01.007 PMC3482120 23109748

[13] Campbell IC, Coudrillier B, Ethier CR. Biomechanics of the posterior eye: a critical role in health and disease. *J Biomechanical Eng*. 2014; 136(2): 021005. 10.1115/1.4026286 24356942

[14] Summers Rada JA, Hollaway LR. Regulation of the biphasic decline in scleral proteoglycan synthesis during the recovery from induced myopia. *Exp Eye Res*. 2011; 92(5): 394–400. 21354134 10.1016/j.exer.2011.02.011 PMC3081968

[15] Seko Y, Shimokawa H, Tokoro T. In vivo and in vitro association of retinoic acid with form-deprivation myopia in the chick. *Exp Eye Res*. 1996; 63(4): 443–452. 8944551 10.1006/exer.1996.0134

[16] Seko Y, Shimizu M, Tokoro T. Retinoic acid increases in the retina of the chick with form deprivation myopia. *Ophthalmic Res*. 1998; 30(6): 361–367. 9731117 10.1159/000055496

[17] Mertz JR, Wallman J. Choroidal retinoic acid synthesis: a possible mediator between refractive error and compensatory eye growth. *Exp Eye Res*. 2000; 70(4): 519–527. 10866000 10.1006/exer.1999.0813

[18] Brown DM, Mazade R, Clarkson-Townsend D, Hogan K, Datta Roy PM, Pardue MT. Candidate pathways for retina to scleral signaling in refractive eye growth. *Exp Eye Res*. 2022; 219: 109071. 35447101 10.1016/j.exer.2022.109071 PMC9701099

[19] Guo L, Frost MR, He L, Siegwart JTJ, Norton TT. Gene expression signatures in tree shrew sclera in response to three myopiagenic conditions. *Invest Ophthalmol Vis Sci*. 2013; 54(10): 6806–6819. 24045991 10.1167/iovs.13-12551 PMC3805087

[20] Guo L, Frost MR, Siegwart JT, Norton TT. Scleral gene expression during recovery from myopia compared with expression during myopia development in tree shrew. *Mol Vis*. 2014; 20: 1643–1659. 25540576 PMC4265769

[21] Guo L, Frost MR, Siegwart JTJ, Norton TT. Gene expression signatures in tree shrew sclera during recovery from minus-lens wear and during plus-lens wear. *Mol Vis*. 2019; 25: 311–328. 31341380 PMC6610222

[22] Li C, McFadden SA, Morgan I, et al. All-trans retinoic acid regulates the expression of the extracellular matrix protein fibulin-1 in the guinea pig sclera and human scleral fibroblasts. *Mol Vis*. 2010; 16: 689–697. 20405022 PMC2855729

[23] McFadden SA, Howlett MHC, Mertz JR. Retinoic acid signals the direction of ocular elongation in the guinea pig eye. *Vis Res*. 2004; 44(7): 643–653. 14751549 10.1016/j.visres.2003.11.002

[24] McFadden SA, Howlett MHC, Mertz JR, Wallman J. Acute effects of dietary retinoic acid on ocular components in the growing chick. *Exp Eye Res*. 2006; 83(4): 949–961. 16797531 10.1016/j.exer.2006.05.002

[25] Cadot S, Frenz D, Maconochie M. A novel method for retinoic acid administration reveals differential and dose-dependent downregulation of Fgf3 in the developing inner ear and anterior CNS. *Dev Dynamics*. 2012; 241(4): 741–758. 10.1002/dvdy.23748 22334445

[26] Feola AJ, Sherwood JM, Pardue MT, Overby DR, Ethier CR. Age and menopause effects on ocular compliance and aqueous outflow. *Invest Ophthalmol Vis Sci*. 2020; 61(5): 16. 10.1167/iovs.61.5.16 PMC7405619 32407519

[27] Jacobs GH, Williams GA, Fenwick JA. Influence of cone pigment coexpression on spectral sensitivity and color vision in the mouse. *Vis Res*. 2004; 44(14): 1615–1622. 15135998 10.1016/j.visres.2004.01.016

[28] Brown DM, Kowalski MA, Paulus QM, et al. Altered structure and function of murine sclera in form-deprivation myopia. *Invest Ophthalmol Vis Sci*. 2022; 63(13): 13. 10.1167/iovs.63.13.13 PMC9753793 36512347

[29] Kane MA, Napoli JL. Quantification of endogenous retinoids. *Methods Mol Biol*. 2010; 652: 1–54. 20552420 10.1007/978-1-60327-325-1_1 PMC4113000

[30] Jones JW, Pierzchalski K, Yu J, Kane MA. Use of fast HPLC multiple reaction monitoring cubed for endogenous retinoic acid quantification in complex matrices. *Anal Chem*. 2015; 87(6): 3222–3230. 25704261 10.1021/ac504597q PMC6761927

[31] Kane MA, Folias AE, Wang C, Napoli JL. Quantitative profiling of endogenous retinoic acid in vivo and in vitro by tandem mass spectrometry. *Anal Chem*. 2008; 80(5): 1702–1708. 18251521 10.1021/ac702030f PMC4086453

[32] Kane MA, Folias AE, Napoli JL. HPLC/UV quantitation of retinal, retinol, and retinyl esters in serum and tissues. *Anal Biochem*. 2008; 378(1): 71–79. 18410739 10.1016/j.ab.2008.03.038 PMC2483537

[33] Faulkner AE, Kim MK, Iuvone PM, Pardue MT. Head-mounted goggles for murine form deprivation myopia. *J Neurosci Methods*. 2007; 161(1): 96–100. 17126909 10.1016/j.jneumeth.2006.10.011

[34] Bergen MA, Park HN, Chakraborty R, et al. Altered refractive development in mice with reduced levels of retinal dopamine. *Invest Ophthalmol Vis Sci*. 2016; 57(10): 4412. 27750284 10.1167/iovs.15-17784 PMC5015967

[35] Schaeffel F, Burkhardt E, Howland HC, Williams RW. Measurement of refractive state and deprivation myopia in two strains of mice. *Optometry Vis Sci*. 2004; 81(2): 99–110. 10.1097/00006324-200402000-00008 15127929

[36] Schaeffel F . Test systems for measuring ocular parameters and visual function in mice. *Front Biosci*. 2008; 13: 4904–4911. 18508555 10.2741/3049

[37] Guizar-Sicairos M, Thurman ST, Fienup JR. Efficient subpixel image registration algorithms. *Optics Lett*. 2008; 33(2): 156–158. 10.1364/ol.33.000156 18197224

[38] Landis E, Chrenek M, Chakraborty R, et al. Increased endogenous dopamine prevents myopia in mice. *Exp Eye Res*. 2020; 193: 107956. 32032629 10.1016/j.exer.2020.107956 PMC7113116

[39] Chakraborty R, Landis EG, Mazade R, et al. Melanopsin modulates refractive development and myopia. *Exp Eye Res*. 2022; 214: 108866. 34838844 10.1016/j.exer.2021.108866 PMC8792255

[40] Brown DM, Pardue MT, Ethier CR. A biphasic approach for characterizing tensile, compressive and hydraulic properties of the sclera. *J R Soc Interface*. 18(174): 20200634. 10.1098/rsif.2020.0634 PMC7879763 33468024

[41] Schindelin J, Arganda-Carreras I, Frise E, et al. Fiji: an open-source platform for biological-image analysis. *Nat Methods*. 2012; 9(7): 676–682. 22743772 10.1038/nmeth.2019 PMC3855844

[42] Bates D, Machler M, Bolker B, Walker S. Fitting linear mixed-effects models using lme4. *J Stat Softw*. 2015; 67(1): 1–48.

[43] Lenth RV . emmeans: estimated marginal means, aka least-squares means. r package version 1.7.0. 2021, https://CRAN.R-project.org/package=emmeans.

[44] Westfall PH, Tobias RD. Multiple testing of general contrasts. *J Am Stat Assoc*. 2007; 102(478): 487–494.

[45] Kane MA, Chen N, Sparks S, Napoli JL. Quantification of endogenous retinoic acid in limited biological samples by LC/MS/MS. *Biochem J*. 2005; 388(pt 1): 363–369. 15628969 10.1042/BJ20041867 PMC1186726

[46] Siegwart JT, Norton TT. Regulation of the mechanical properties of tree shrew sclera by the visual environment. *Vis Res*. 1999; 39(2): 387–407. 10326144 10.1016/s0042-6989(98)00150-3

[47] Phillips JR, Khalaj M, McBrien NA. Induced myopia associated with increased scleral creep in chick and tree shrew eyes. *Invest Ophthalmol Vis Sci*. 2000; 41(8): 2028–2034. 10892839

[48] Lewis JA, Garcia MB, Rani L, Wildsoet CF. Intact globe inflation testing of changes in scleral mechanics in myopia and recovery. *Exp Eye Res*. 2014; 127: 42–48. 25041940 10.1016/j.exer.2014.07.004 PMC4174731

[49] Schmucker C, Schaeffel F. In vivo biometry in the mouse eye with low coherence interferometry. *Vis Res*. 2004; 44(21): 2445–2456. 15358080 10.1016/j.visres.2004.05.018

[50] Schmucker C, Schaeffel F. A paraxial schematic eye model for the growing C57BL/6 mouse. *Vis Res*. 2004; 44(16): 1857–1867. 15145680 10.1016/j.visres.2004.03.011

[51] Tkatchenko TV, Shen Y, Tkatchenko AV. Mouse experimental myopia has features of primate myopia. *Invest Ophthalmol Vis Sci*. 2010; 51(3): 1297–1303. 19875658 10.1167/iovs.09-4153 PMC2829371

[52] Schaeffel F, Howland HC, Farkas L. Natural accommodation in the growing chicken. *Vis Res*. 1986; 26(12): 1977–1993. 3617538 10.1016/0042-6989(86)90123-9

[53] Howlett MHC, McFadden SA. Emmetropization and schematic eye models in developing pigmented guinea pigs. *Vis Res*. 2007; 47(9): 1178–1190. 17360016 10.1016/j.visres.2006.12.019

[54] Summers JA . *Retinoic Acid in Ocular Growth Regulation*. IntechOpen; 2019, doi:10.5772/intechopen.84586.

[55] Moring AG, Baker JR, Norton TT. Modulation of glycosaminoglycan levels in tree shrew sclera during lens-induced myopia development and recovery. *Invest Ophthalmol Vis Sci*. 2007; 48(7): 2947–2956. 17591859 10.1167/iovs.06-0906 PMC2080847

[56] Levick JR . Flow through interstitium and other fibrous matrices. *Q J Exp Physiol*. 1987; 72(4): 409–437. 3321140 10.1113/expphysiol.1987.sp003085

[57] Quinn TM, Dierickx P, Grodzinsky AJ. Glycosaminoglycan network geometry may contribute to anisotropic hydraulic permeability in cartilage under compression. *J Biomechanics*. 2001; 34(11): 1483–1490. 10.1016/s0021-9290(01)00103-8 11672723

[58] Farndale RW, Sayers CA, Barrett AJ. A direct spectrophotometric microassay for sulfated glycosaminoglycans in cartilage cultures. *Connective Tissue Res*. 1982; 9(4): 247–248. 10.3109/03008208209160269 6215207

[59] Rada JA, Thoft RA, Hassell JR. Increased aggrecan (cartilage proteoglycan) production in the sclera of myopic chicks. *Dev Biol*. 1991; 147(2): 303–312. 1916012 10.1016/0012-1606(91)90288-e

