# Supplementary material for: Exogenous All-Trans Retinoic Acid Induces Myopia and Alters Scleral Biomechanics in Mice
Source: Invest Ophthalmol Vis Sci. 2023 May 23;64(5):22. doi: 10.1167/iovs.64.5.22 (PMC10210516; doi:10.1167/iovs.64.5.22)
Supplement: Supplement 1 [file iovs-64-5-22_s001.pdf]

Exogenous all-trans retinoic acid induces myopia and alters scleral biomechanics in mice

Brown et al., IOVS 2023

Supplementary Data

Table S.1: Summary of statistical models used to analyze study outcomes. Covariate "days deviation" adjusts for deviations in age at the time of a measurement. "*": full interaction operator (indicating both main effects and their interaction), "1|animal id": random intercept for each animal. UCT: unconfined compression test; DMMB: dimethylmethylene blue assay; PG: picogreen assay; IHC: immunohistochemistry; LC-MS/MS: Liquid chromatography tandem mass spectrometry; DVs: Dependent variables; RE: Refractive error; AL: Axial length; H_{+A}: Aggregate tensile modulus (tensile stiffness); k: hydraulic conductivity (permeability); GAG: glycosaminoglycan; atRA: all-trans retinoic acid; tRE: total retinyl esters; ROL: Retinol.

Outcomes	DVs	Fixed Effects	Covariates	Random Effects	Family	Link
**In Vivo**	RE, AL, etc.	~ timepoint*treatment	days_deviation	1|animal_id	Gaussian	Identity
**LCMSMS**	atRA, tRE, ROL	~ treatment*tissue			Gaussian	Identity
**UCT**	H_{+A}, k	~ treatment*strain		1|animal_id	Gamma	Log
**DMMB/PG**	GAG/mass, GAG/DNA	~ treatment		1|animal_id	Gamma	Log
**IHC**	NormIntensity	~ treatment		1|animal_id	Gamma	Log

Table S.2: Raw ocular biometry at each timepoint of the study. Groups are presented as "Group (# animals)". Tabulated data are the mean ± standard deviation of the outcome. Bolded values are significantly different from the controls at the same timepoint (p<0.05), using the regression models and adjusting for deviations in age. RE: Refractive error; CC: Corneal curvature; CCT: central corneal thickness; ACD: Anterior chamber depth; LT: Lens thickness; VCD: Vitreous chamber depth; AL: Axial length; Ctrl: Vehicle-treated group; RA: atRA-treated group.

Timepoint	Group (n)	RE [D]	CC [mm]	CCT [µm]	ACD [µm]	LT [mm]	VCD [µm]	RT [µm]	AL [mm]
**Baseline**	Ctrl (14)	0.90±0.87	1.41±0.01	92.80±3.06	335.99±13.91	1.78±0.01	669.57±21.59	181.71±9.28	3.09±0.03
	RA (16)	1.02±1.05	1.40±0.02	**95.36±3.51**	338.49±11.74	1.78±0.02	670.15±24.40	184.25±7.06	3.10±0.03
**1 Week**	Ctrl (14)	4.88±0.65	1.46±0.02	95.60±2.21	354.99±10.85	1.87±0.02	645.46±6.02	176.98±7.08	3.17±0.02
	RA (16)	**1.17±2.20**	1.46±0.02	96.39±3.28	347.96±12.78	1.87±0.02	**666.18±15.14**	176.16±5.33	3.19±0.04
**2 Weeks**	Ctrl (14)	6.45±0.43	1.49±0.02	94.61±2.53	370.67±11.76	1.92±0.02	625.71±15.16	175.54±4.80	3.22±0.02
	RA (16)	**0.74±2.20**	1.48±0.01	94.49±3.72	363.01±12.04	1.92±0.03	**657.99±24.83**	173.01±10.26	3.24±0.03

Table S.3: Change from baseline over the two weeks of treatment in ocular biometry. Groups are presented as "Group (# animals)". Tabulated data are the mean ± standard deviation of the change from baseline (at time t_0) of each animal ($\Delta^t x_i$; $x_i^{t_n} - x_i^{t_0}$), where $x_i^{t_n}$ represents a measured quantity in animal i at timepoint t_n. Bolded values are significantly different from the controls at the same timepoint ($p<0.05$), using the regression models and adjusting for deviations in age. RE: Refractive error; CC: Corneal curvature; CCT: central corneal thickness; ACD: Anterior chamber depth; LT: Lens thickness; VCD: Vitreous chamber depth; AL: Axial length; Ctrl: Vehicle-treated group; RA: atRA-treated group.

Timepoint	Group (n)	RE [D]	CC [µm]	CCT [µm]	ACD [µm]	LT [µm]	VCD [µm]	RT [µm]	AL [µm]
1 week	Ctrl (14)	3.98±0.97	56.85±12.28	3.01±3.58	19.86±7.45	89.05±25.12	-25.16±24.19	-4.84±8.60	82.58±14.72
	RA (16)	**0.14±1.57**	59.06±16.73	0.61±3.25	**11.13±8.78**	86.74±24.14	**-2.16±25.80**	-6.85±6.75	**90.86±17.45**
2 week	Ctrl (14)	5.55±1.02	82.28±15.13	2.21±3.51	35.68±9.13	144.91±30.33	-45.08±32.07	-6.37±8.50	132.05±15.14
	RA (16)	**-0.27±1.64**	82.71±17.18	0.02±4.11	**26.69±7.12**	143.36±28.67	**-18.17±37.29**	-7.59±7.53	**145.01±16.64**

Table S.4: Material properties of the sclera obtained from unconfined compression. Groups are presented as "Group (# eyes)". Tabulated values are the mean ± standard deviation of unadjusted, fitted material properties at each step (5%, 10%, 15% compressive strain) and the average over the steps. Bolded values are significantly different from the controls ($p<0.05$), using the regression models and adjusting for deviations in applied compressive strain. H_{+A}: Aggregate in-plane tensile modulus (stiffness); k: Hydraulic conductivity (permeability); RA: atRA-treated animals; Ctrl: vehicle-treated animals.

	H_{+A} [kPa]				k $[m^4/Pa \cdot s]$ (x10^{14})			
**Group (# eyes)**	**Step 1**	**Step 2**	**Step 3**	**Average**	**Step 1**	**Step 2**	**Step 3**	**Average**
**Ctrl (8)**	79.42±13.70	131.81±33.02	269.12±83.27	160.12±95.79	1.60±0.88	0.45±0.28	0.15±0.06	0.73±0.82
**RA (10)**	64.34±14.32	**94.94±21.12**	**152.29±46.99**	**103.86±47.56**	1.89±1.04	**0.68±0.25**	**0.33±0.20**	**0.96±0.91**

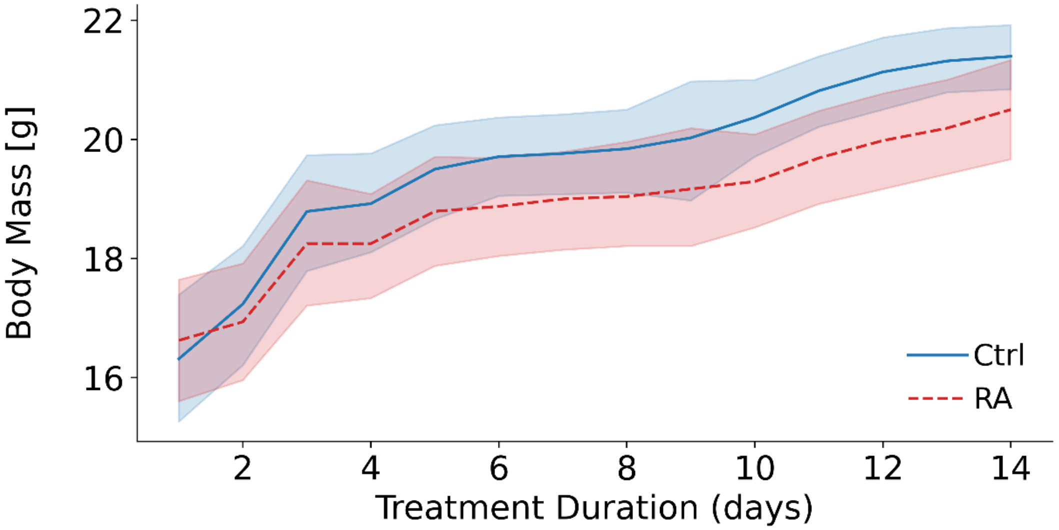

Figure S.1: Body mass was not significantly affected by two-weeks of atRA treatment. Lines are the mean body masses of the control (solid, blue) and atRA-treated (dashed, red) animals. Shaded regions show the 95% confidence intervals. Ctrl: Vehicle-treated group (n=14 animals); RA: atRA-treated group (n=16 animals).

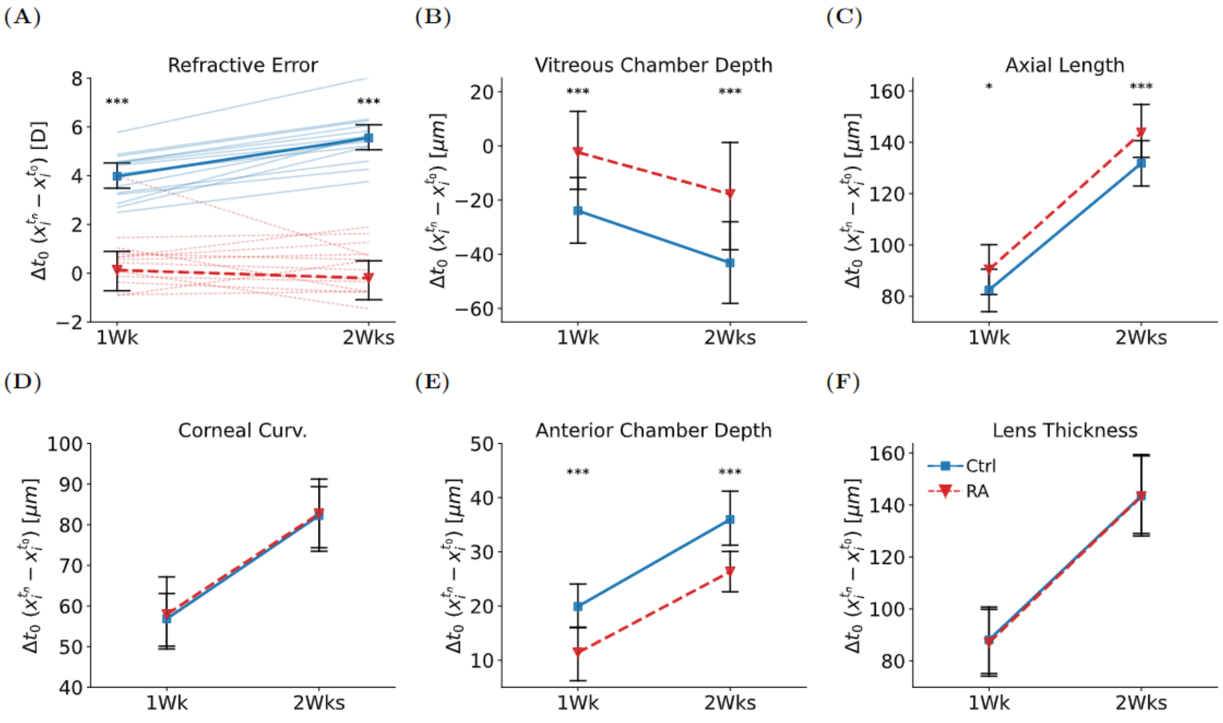

Figure S.2: Treatment with atRA significantly altered the development of refractive state and ocular biometry. Plots show the changes from baseline (t_0) of each outcome ($\Delta^t x_i$; ${x_i}^{t_n} - {x_i}^{t_0}$), where ${x_i}^{t_n}$ represents the measured quantity in animal i at timepoint t_n. (A) Development of refractive error was significantly influenced by atRA, with 1 and 2 weeks of daily atRA causing significant myopia to develop relative to the control animals.

(B) VCD and (C) axial length increased more over the treatment period in the atRA-treated animals vs. controls. (D-F) Biometry of the anterior eye was largely unaffected, except for a small shrinkage of the anterior chamber. Individual animals are shown with fainter lines in (A). Solid (blue) lines are the mean of the control group, dashed (red) lines are the mean of the atRA-treated group. Error bars show the 95% confidence intervals. Ctrl: Vehicle-treated group (n=14 animals); RA: atRA-treated group (n=16 animals). *: $p<0.05$, **: $p<0.01$, ***: $p<0.001$, adjusting for deviations in age and multiple comparisons.

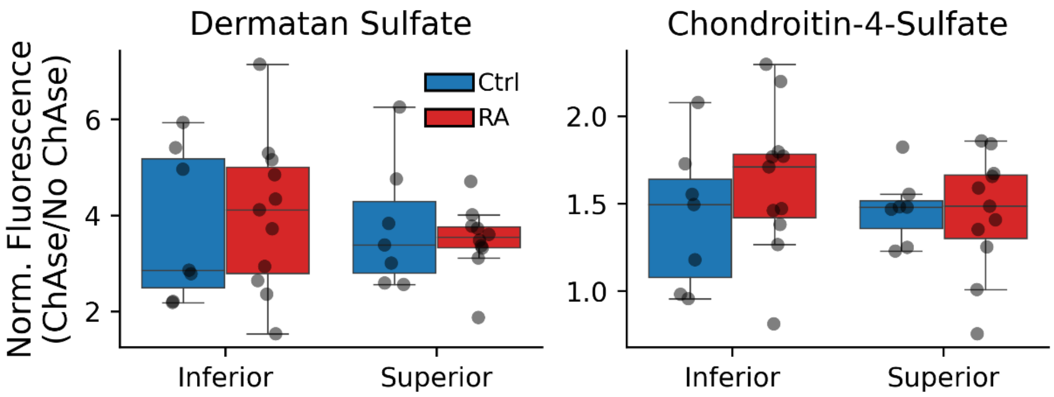

Figure S.3: Immunolabeling of glycosaminoglycans was not measurably changed by atRA treatment or different between the superior and inferior sclera.
